# Discovery of 4,5-Dihydro-1*H*-thieno[2′,3′:2,3]thiepino [4,5-c]pyrazole-3-carboxamide Derivatives as the Potential Epidermal Growth Factor Receptors for Tyrosine Kinase Inhibitors

**DOI:** 10.3390/molecules23081980

**Published:** 2018-08-08

**Authors:** Jia Ke, Qi Lu, Xin Wang, Rui Sun, Zhe Jin, Xiaoyi Zhan, Jianshu Hu, David Chi-cheong Wan, Chun Hu

**Affiliations:** 1 Key Laboratory of Structure-Based Drug Design & Discovery, Ministry of Education, Shenyang Pharmaceutical University, Shenyang 110016, China; kejiaRejoice815@139.com (J.K.); xiaoliyuyouxiang@163.com (Q.L.); xinw521@163.com (X.W.); sunruisun@163.com (R.S.); xiaoyizhan1996@163.com (X.Z.); 2 School of Biomedical Sciences, Faculty of Medicine, The Chinese University of Hong Kong, Hong Kong, China; hujianshu999@gmail.com (J.H.); chicheongwan@cuhk.edu.hk (D.C.-c.W.)

**Keywords:** heterocycle, synthesis, EGFR, tyrosine kinase inhibitor

## Abstract

The epidermal growth factor receptors (EGFRs), in which overexpression (known as upregulation) or overactivity have been associated with a number of cancers, has become an attractive molecular target for the treatment of selective cancers. We report here the design and synthesis of a novel series of 4,5-dihydro-1*H*-thieno [2′,3′:2,3]thiepino[4,5-c]pyrazole-3-carboxamide derivatives and the screening for their inhibitory activity on the EGFR high-expressing human A549 cell line using 3-(4,5-dimethylthiazol-2-yl)-2,5-diphenyltetrazolium bromide (MTT). A Docking simulation was performed to fit compound **6g** and gifitinib into the EGFR to determine the probable binding models, and the binding sites and modes conformation of **6g** and gifitinib were exactly similar, the two compounds were stabilized by hydrogen bond interactions with MET769. Combining with the biological activity evaluation, compound **6g** demonstrated the most potent inhibitory activity (IC_50_ = 9.68 ± 1.95 μmol·L^–1^ for A549). Conclusively, 4,5-dihydro-1*H*-thieno[2′,3′:2,3]thiepino[4,5-c]pyrazole-3-carboxamide derivatives as the EGFR tyrosine kinase inhibitors were discovered, and could be used as potential lead compounds against cancer cells.

## 1. Introduction

The epidermal growth factor receptor (EGFR) is the cell-surface receptor for members of the epidermal growth factor family of extracellular protein ligands [1]. The over expression and/or mutation of EGFR tyrosine kinase has been observed in many human solid tumors, including non-small cell lung, breast, ovarian and squamous cell cancers [2,3,4,5], EGFR has been under intense investigation as a novel anticancer molecular target [6]. Small molecular inhibitors that attenuate the ability of EGFR to transmit a phosphorylation signal following the binding of its cognate ligand EGF have become an important class of potential anticancer drugs [7]. As it is known, three 4-(phenylamino)quinazoline derivatives, gefitinib (CP358774), erlotinib (ZD 1839) and lapatinib (GW572016) have been approved by FDA, and their analogs are being studied extensively to circumvent the resistance while improving the drug efficacy. Now new drug candidates in this class have already reached various phases of clinical trials [8,9].

Based on the principles of bioisosterism, structural optimization, and on the combination of the active substructures of EGFR inhibitors which have been reported, a novel series of 4,5-dihydro-1*H*-thieno [2′,3′:2,3]thiepino[4,5-c]pyrazole-3-carboxamide derivatives was designed using molecular docking and subsequently synthesized in satisfactory yields.

We described here the synthesis and the structure-activity relationship of the novel series of 4,5-dihydro-1*H*-thieno [2′,3′:2,3]thiepino[4,5-c]pyrazole-3-carboxamide derivatives, and tested them for their inhibitory activity on the EGFR high-expressing human A549 cell line using the MTT cell viability assay. The screening results showed that compound **6g** is the most potent inhibitor (IC_50_ = 9.68 ± 1.95 for EGFR), which was compared with the positive control gifitinib. A preliminary structure-activity relationships of the target compounds were summarized.

In order to investigate the interaction between the target compounds and the target proteins, docking simulations were performed using the X-ray crystallographic structure of the EGFR in complex with a compound to explore the binding modes of these compounds at the active site. Compound **6g** and the positive control gefitinib were superimposed onto the crystal structure of erlotinib in EGFR (PDB: 4HJO) [10] using Molegro Virtual Docker (2008 v3.0). Molecular modeling showed that the hydrogen-bonds were detected between compound **6g**, gefitinib, and MET769, and the residues LEU694, THR766, GLN767, and LEU 768 formed some weak interactions with the two compounds.

## 2. Results and Discussion

### 2.1. Synthesis

**Scheme 1:**
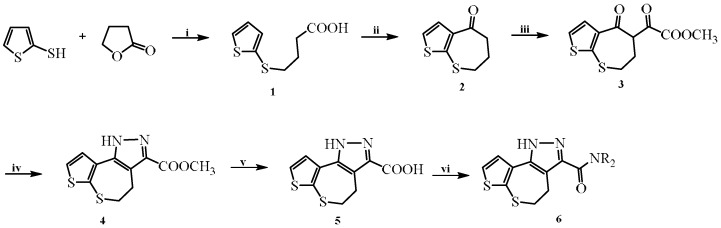
The synthesis of 4,5-dihydro-1*H*-thieno [2′,3′:2,3]thiepino[4,5-c]pyrazole-3-carboxamide derivatives. (i) sodium ethoxide/C_2_H_5_OH, reflux, N_2_; then HCl; (ii) oxalyl chloride, r.t, 1 h, N_2_; then SnCl_4_, 0 °C, 12 h, N_2_; (iii) sodium methoxide, dimethyl oxalate, room temperature (r.t), 24 h; (iv) 80% hydrazine hydrate, reflux, 4 h; (v) NaOH/H_2_O, reflux; 2/18% HCl; (vi) 1­substituted piprazine or aliphatic cycloamine, EDCI/HOBt, Et_3_N, r.t. 24 h.

In general, the target compounds (**6a**–**6e**) were obtained in satisfactory yields, and the synthetic pathways are described in Scheme 1.

**4**-(Thiophen-**2**-ylthio)butanoic acid (**1**) was obtained by treating **2**-mercaptothiophene with γ-butyrolactone under the nitrogen gas, which was reacted with oxalyl chloride and tin tetrachloride under nitrogen gas to give **2** [11]. The reaction of **2** with dimethyl oxalate in dry toluene at room temperature for 24 h in the presence of sodium hydride gave rise to the compound **3** [12]. A mixture of **3** and hydrazine hydrate in acetic acid was then refluxed for 4 h to afford **4** [13], which upon treatment with first NaOH in water and then 18% hydrochloric acid afforded **5**. Target compounds **6a**–**6l** were obtained by reacting **5** with piperazine derivatives or aliphatic cycloamines in the presence of EDCI and HOBt and were purified by column chromatography on silica gel with a mixture of ethyl acetate/petroleum ether as the eluent. The structures of all the target compounds were confirmed by infrared spectra, proton nuclear magnetic resonance spectroscopy (NMR), carbon NMR, and mass spectrometries including high resolution mass spectrometry, and these compounds gave satisfactory analytical and spectroscopic data, which were in full accordance with their depicted structures.

### 2.2. Biological Evaluation

The potencies for inhibition of target compounds **6a**–**6l** were evaluated on the EGFR high-expressing human lung adenocarcinoma cell line A549 [14] and EGFR low-expressing human liver cancer cell line HepG2 [15,16,17], using the MTT colorimetric assay. The calculated IC_50_ value is the concentration (mu-gram/mL) of a compound which was able to cause 50% growth inhibition with respect to the control culture, was reported in Table 1.

In general, the screening results showed that most of the target compounds exhibit the remarked antiproliferative activities against A549 cells, and all the target compounds exhibit hardly any antiproliferative activities against HepG2 cells. On the whole, the antiproliferative activities of the compounds with aliphatic cycloamino group including pyrrolidin-1-yl, piperidin-1-yl, and morpholin-4-yl are stronger than that of the compounds with piperazin-1-yl groups, whereas the target compounds with piperazin-1-yl groups appeared a great fluctuation associated with the variety of substituents in the antiproliferative activities against A549 cells. As shown in Table 1, compound **6g** displayed the most potent inhibitory activity (IC_50_ = 9.68 ± 1.95 μmol·L^−1^), comparable to the positive control gifitinib (IC_50_ = 8.58 ± 1.65 μmol·L^−1^). When methyl and methoxy (**6c** and **6d**) displaced the hydrogen at 4-positions of the phenyl ring, the inhibitory activity enhanced compared to **6b** which has no substituent on the phenyl ring, but the inhibitory activity disappeared when this place was displaced by electron-withdrawing group such as fluoro (**6e**). As for the compounds with piperazin-1-yl groups, ortho-substitution with methyl (**6f**) or fluoro (**6g**) also enhanced the activity compared to compound **6b**. Preliminary structure-activity relationships in these derivatives demonstrated that compounds with substituents at the ortho position and electron-donating group at para position of phenyl ring would show more potent activities. There is no obvious difference between **6a** and **6b**. However, when the hydrogen of methyl was substituted with two phenyl instead of just hydrogen (**6i** vs. **6a**), the potency of the compound decreased remarkably. This result indicated that small substitution at nitrogen of piperazine is preferred over bulky (phenyl) substitutions.

### 2.3. Docking Study

A docking study aiming at the characterization of the interactions between the target molecule and EGFR at molecular level was carried out.

**Figure 1:**
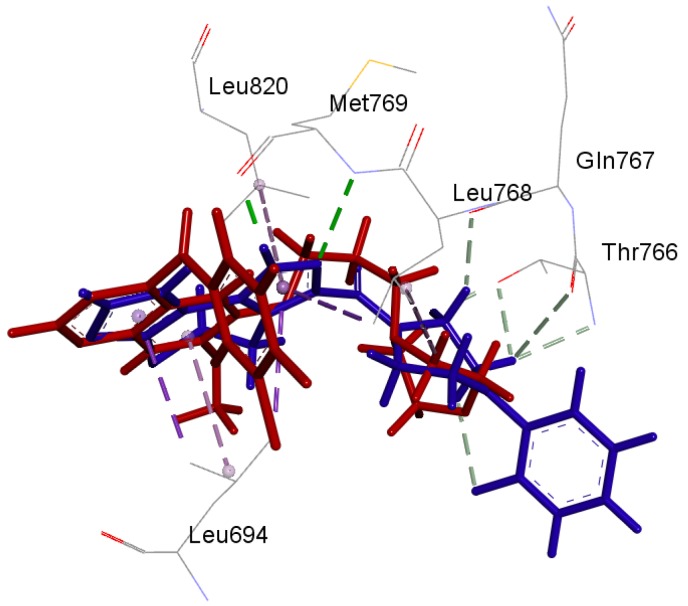
Interactions of **6g** (blue) and gefitinib (red) with epidermal growth factor receptor (EGFR) in a three-dimensional diagram.

**Figure 2:**
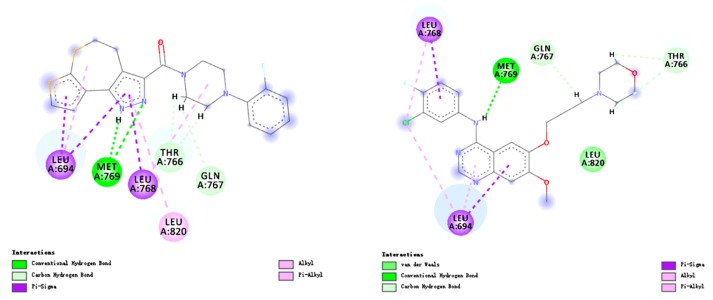
Interactions of **6g** (left) and Gefitinib (right) with EGFR in two-dimensional diagram.

In this study, the model was selected from Protein Data Bank of Research Collaboratory for Structural Bioinformatics (RCSB PDB), and the docking study was prepared by Molegro Virtual Docker (2008 v3.0). Initially, the docking studies were carried out on the EGFR-erlotinib complex (PDB code 4HJO) [10]. The target compound **6g** with a fluorine atom at 4-positions of phenyl ring and the positive control gefitinib were docked to the crystal structure EGFR, as shown in Figure 1 and Figure 2.

In the binding model, compound **6g** and the positive control gefitinib were docked nicely to the protein. The amino acid residue MET769 formed hydrogen bonds with **6g** and gefitinib respectively, and the residues LEU694, THR766, GLN767, and LEU 768 all formed some weak interactions with the two compounds.

The molecular docking result showed that compound **6g** was a potential inhibitor of EGFR and its analogs might have similar effect. So we synthesized a series of 4,5-dihydro-1*H*-thieno [2′,3′:2,3]thiepino[4,5-c]pyrazole-3-carboxamide derivatives aiming to test their inhibitory activity to EGFR.

## 3. Experimental Section

### 3.1. Chemistry

Melting points were determined on a XT4 MP apparatus (Taike Corp., Beijing, China) and were uncorrected. The reactions were monitored by thin-layer chromatography (TLC) with silica gel GF254 plates, which were visualized by ultraviolet (UV) light (254 nm). ^1^H-NMR and ^13^C NMR spectra were measured on a Bruker 600 MHz and 400 MHz spectrometer at 25 °C respectively, and referenced to Me_4_Si. Chemical shifts are reported in ppm (δ) using the residual solvent line as an internal standard. Splitting patterns are designed as s, singlet; d, doublet; t, triplet; m, multiple. Electrospray ionization-mass spectrometry (ESI-MS) spectra were recorded on a Waters spectrometer. The infrared (IR) spectra were obtained using a Bruker IFS55 spectrometer. High-resolution mass spectral (HRMS) analyses were recorded on a Bruker FT MS SOLARIX 7.0T Mass Spectrometer with a Fourier-transform-ion-cyclotron-resonance-mass-spectrometer (FT-ICR-MS)-equipped ESI source. Starting materials and solvents were purchased from common commercial suppliers and were used without further purification.

#### 3.1.1. Synthesis of 4-(Thiophen-2-ylthio)butanoic Acid (**1**)

The sodium ethoxide was prepared with sodium (3.34 g, 145 mmol) and ethanol (120 mL). 2-mercaptothiophene (9.80 g, 84 mmol) was added dropwise to this solvent and stirred 30 min at room temperature. γ-Butyrolactone (11.5 mL, 128 mmol) was added and the mixture was refluxed for 19 h under N_2_. The mixture was concentrated and the residue was solved in water. The solution was acidified with 18% hydrochloric acid to pH 1–2, and then extracted three times with EtOAc. The combined organic layer was dried, filtered, and concentrated. The residue was recrystallized from petroleum ether to give **1** (8.08 g, 47.4%) as a white solid. m.p. 40–42 °C.

#### 3.1.2. Synthesis of 6,7-Dihydrothieno[2,3-*b*]thiepin-4(*5H*)-one (**2**)

To a solution of **1** (4.04 g, 20 mmol) and **2** drops of DMF in CH_2_Cl_2_ (20 mL), oxalyl chloride (2.81 g, 22 mmol) was added dropwise at room temperature under N_2_. After the mixture was stirred for 1 h, it was cooled to 0 °C and a solution of SnCl_4_ (2.57 g, 10 mmol) in CH_2_Cl_2_ (10 mL) was added dropwise. The solution was stirred at 0 °C for 12 h, then H_2_O was added. The organic layer was separated and washed with saturated Na_2_CO_3_ and brine respectively. After drying and filtration, the resulting organic solution was concentrated to dryness under vacuum. The residue was recrystallized from petroleum ether to yield **2** (0.92 g, 25.0%) as a white solid. Melting point (m.p.): 60–62 °C, ^1^H-NMR (600 MHz, CDCl_3_): δ 2.25–2.28 (m, 2H), 3.02 (t, 2H, *J* = 5.4 Hz), 3.06 (t, 2H, *J* = 5.4 Hz), 7.08 (d, 1H, *J* = 5.4 Hz), 7.43 (d, 1H, *J* = 5.4 Hz).

#### 3.1.3. Synthesis of Methyl Oxo(4-oxo-4,5,6,7-tetrahydrothieno[2,3-*b*]thiepin-5-yl)acetate (**3**)

The sodium methoxide was prepared with sodium (0.46 g, 20 mmol) and methanol (20 mL) which was removed by distillation when sodium was dissolved completely. A solution of **2** (1.84 g, 10 mmol) and dimethyl oxalate (2.36 g, 20 mmol) in dry toluene (20 mL) was added to the sodium methoxide. The mixture was stirred for 24 h at room temperature. Then the mixture was poured into 100 mL water. The aqueous layer was separated, and the organic layer was extracted three times with 10% NaOH (each 15 mL). The combined aqueous layer was acidified with 18% hydrochloric acid to deposit **3** (1.62 g, 60.0%) as a red oil.

#### 3.1.4. Synthesis of Methyl 4,5-dihydro-1*H*-thieno[2′,3′:2,3]thiepino[4,5-*c*] pyrazole-3-carboxylate (**4**)

A solution of **3** (0.54 g, 2 mmol) and 80% hydrazine hydrate (2 mL, 32 mmol) in acetic acid (10 mL) was refluxed for 4 h. The reaction mixture was poured into ice-water mixture (50 g) and allowed to stand overnight. A precipitate was collected to afford **4** (0.40 g, 75.0%) as a white solid. m.p. 98–100 °C. ^1^H-NMR (600 MHz, CDCl_3_): δ 3.10 (t, 2H, *J* = 6.0 Hz), 3.69 (t, 2H, *J* = 6.0 Hz), 3.97 (s, 3H), 7.21 (d, 1H, *J* = 5.4 Hz), 7.24 (d, 1H, *J* = 5.4 Hz).

#### 3.1.5. Synthesis of 4,5-Dihydro-1*H*-thieno[2′,3′:2,3]thiepin[4,5-*c*]pyrazole-3-carboxylic acid (**5**)

To a suspension of **4** (5.32 g, 20 mmol) in 300 mL water, NaOH (1.0 g, 25 mmol) was added. The mixture was refluxed till all solids disappeared, then cooled to room temperature. Subsequently, the solution was acidified with 18% hydrochloric acid until no precipitation appeared. The precipitation was filtered and dried to give **5** (4.54 g, 90.0%) as a white solid. m.p. 166.0–167 °C. ^1^H-NMR (600 MHz, DMSO­*d*_6_): δ 3.10 (t, 2H, *J* = 6.0 Hz), 3.69 (t, 2H, *J* = 6.0 Hz), 7.27 (d, 1H, *J* = 5.4 Hz), 7.62 (d, 1H, *J* = 5.4 Hz), 13.13 (s, 1H).

#### 3.1.6. General Procedure for the Synthesis of 4,5-Dihydro-1*H*-thieno[2′,3′:2,3]thiepino[4,5-c]pyrazole-3-carboxamides (**6a**–**6l**)

A mixture of **5** (1.01 g, 4 mmol), piperazine derivative (4 mmol), **1**­ethyl-**3**-(**3**­dimethylaminopropyl)carbodiimide hydrochloride (1.00 g, 5 mmol), **1**­hydroxybenzotriazole (0.10 g, 0.7 mmol), 1 mL Et_3_N and absolutely dry CH_2_Cl_2_ (20 mL) was stirred for 24 h at room temperature. The mixture was filtered and the filtrate was washed with 20 mL of 1N HCl, saturated Na_2_CO_3_ and brine respectively. Then the organic layer was dried, filtered, and concentrated. The residue was purified by column chromatography on silica with EtOAc-petroleum (3:1, *v*/*v*) as the eluent to produce the product as a white solid.

*1-(4,5-Dihydro-1H-thieno[2′,3′:2,3]thiepino[4,5-c]pyrazole-3-carbonyl)-4-methylpiperazine* (**6a**): White powder; yield: 45.0%; mp: 107–108 °C; IR (KBr, cm^−1^): υ 3429.0, 3228.4, 2915.6, 2792.8, 1608.8, 1504.8, 1439.5, 1352.5, 1290.1, 1253.7, 1225.3, 1142.0, 997.9, 861.7, 766.6; ^1^H-NMR (600 MHz, CDCl_3_): δ 2.33 (s, 3H), 2.42–2.50 (m, 4H), 3.10 (t, 2H, *J* = 6.0 Hz), 3.46 (t, 2H, *J* = 6.0 Hz), 3.76–3.82 (m, 4H), 7.23 (d, 1H, *J* = 5.4 Hz), 7.26 (d, 1H, *J* = 5.4 Hz); ^13^C NMR (101 MHz, DMSO-*d*_6_): δ162.93, 132.23, 131.58, 129.35, 127.16, 126.26, 126.17, 116.44, 55.25, 54.51, 46.63, 45.70, 41.39, 32,25, 29.44; ESI-MS: *m*/*z* 335.2 ([M + H]^+^); HRMS (ESI): *m*/*z* 335.099830 ([M + H]^+^), 357.082060 ([M + Na]^+^).

*1-(4,5-Dihydro-1H-thieno[2′,3′:2,3]thiepino[4,5-c]pyrazole-3-carbonyl)-4-phenylpiperazine* (**6b**): White powder; yield: 30.0%; mp: 174–175 °C; IR (KBr, cm^−1^): υ 3441.7, 3209.2, 2920.5, 2851.6, 1599.6, 1495.6, 1444.2, 1384.1, 1260.4, 1230.6, 1153.3, 1016.0, 998.3, 875.4, 759.5; ^1^H-NMR (600 MHz, CDCl_3_): δ 3.10 (t, 2H, *J* = 6.0 Hz), 3.20 (t, 1H, *J* = 6.0 Hz), 3.24–3.28 (m, 4H), 3.47 (t, 1H, *J* = 6.0 Hz), 4.01–4.05 (m, 4H), 6.90–6.93 (m, 1H), 6.94–6.97 (m, 2H), 7.23 (d, 1H, *J* = 5.4 Hz), 7.28 (d, 1H, *J* = 5.4 Hz), 7.28–7.29 (m, 2H); ^13^C NMR (101 MHz, DMSO-*d*_6_): δ 161.95, 150.91, 140.88,135.37, 129.06, 128.84, 128.74, 125.34, 124.44, 122.66, 119.39, 116.19, 115.94, 115.82, 49.19, 48.54, 46.33, 41.74, 35.62, 24.19; ESI-MS: *m*/*z* 397.2 ([M + H]^+^); HRMS (ESI): 397.115349 ([M + H]^+^), 419.097610 ([M + Na]^+^).

*1-(4,5-Dihydro-1H-thieno[2′,3′:2,3]thiepino[4,5-c]pyrazole-3-carbonyl)-4-(4-methylphenyl)piperazine* (**6c**): White powder; yield: 28.0%; mp: 186–187 °C; IR (KBr, cm^−1^): υ 3124.2, 2916.4, 1631.5, 1579.3, 1513.5, 1443.3, 1384.4, 1340.9, 1264.6, 1236.5, 1203.3, 1154.1, 1016.6, 999.2, 876.2, 811.3; ^1^H-NMR (600 MHz, CDCl_3_): δ 2.28 (s, 3H), 3.10 (t, 2H, *J* = 6.0 Hz), 3.14–3.20 (m, 4H), 3.47 (t, 2H, *J* = 6.0 Hz), 3.91–3.96 (m, 4H), 6.85 (d, 2H, *J* = 8.4 Hz), 7.10 (d, 2H, *J* = 8.4 Hz), 7.23 (d, 1H, *J* = 5.4 Hz), 7.27 (d, 1H, *J* = 5.4 Hz); ESI-MS: *m*/*z* 411.2 ([M + H]^+^); HRMS (ESI): 411.131408 ([M + H]^+^), 433.113470 ([M + Na]^+^).

*1-(4,5-Dihydro-1H-thieno[2′,3′:2,3]thiepino[4,5-c]pyrazole-3-carbonyl)-4-(4-methoxyphenyl)piperazine* (**6d**): White powder; yield: 33.0%; mp: 156–158 °C; IR (KBr, cm^−1^): υ 3443.8, 3196.6, 2920.6, 2851.1, 1611.7, 1510.5, 1445.2, 1384.2, 1279.1, 1246.5, 1225.8, 1154.6, 1034.6, 995.2, 859.4; ^1^H-NMR (600 MHz, CDCl_3_): δ 3.09–3.12 (m, 10H), 3.47 (t, 2H, *J* = 6.0 Hz), 3.78 (s, 3H), 6.85 (d, 2H, *J* = 9.0 Hz), 6.92 (d, 2H, *J* = 9.0 Hz), 7.24 (d, 1H, *J* = 4.8 Hz), 7.32 (d, 1H, *J* = 4.8 Hz); ^13^C NMR (101 MHz, DMSO-*d*_6_): δ 163.43, 153.80, 145.95, 145.62, 135.49, 132.89, 131.42, 127.26, 126.80, 118.54, 117.20, 114.76, 55.65, 51.17, 50.49, 47.14, 45.84, 41.92, 32.63, 29.83; ESI-MS: *m*/*z* 427.2 ([M + H]^+^), 449.2 ([M + Na]^+^); HRMS (ESI): 427.126855 ([M + H]^+^), 449.109060 ([M + Na]^+^).

*1-(4,5-Dihydro-1H-thieno[2′,3′:2,3]thiepino[4,5-c]pyrazole-3-carbonyl)-4-(4-fluorophenyl)piperazine* (**6e**): White powder; yield: 26.0%; mp: 195–197 °C; IR (KBr, cm^−1^): υ 3441.9, 2919.7, 2851.3, 1603.7, 1509.0, 1444.9, 1384.5, 1341.8, 1263.2, 1229.1, 1157.7, 1017.4, 999.5, 877.7, 817.2; ^1^H-NMR (600 MHz, CDCl_3_): δ 3.11 (t, 2H, *J* = 6.0 Hz), 3.27–3.31 (m, 4H), 3.50 (t, 2H, *J* = 6.0 Hz), 3.96–4.00 (m, 4H), 6.91 (dd, 2H, *J* = 9.1 Hz, 4.2 Hz), 6.99 (dd, 2H, *J* = 9.1 Hz, 4.2 Hz), 7.24 (d, 1H, *J* = 5.4 Hz), 7.25 (d, 1H, *J* = 5.4 Hz); ^13^C NMR (101 MHz, DMSO-*d*_6_): δ 161.95, 157.55, 155.20, 147.80, 135.60, 128.78, 125.42, 124.42, 122.68, 117.85, 117.77, 116.14, 115.53, 115.31, 49.96, 49,43, 46.39, 41.76, 35.55, 24.14; ESI-MS: *m*/*z* 415.1 ([M + H]^+^); HRMS (ESI): 415.106539 ([M + H]^+^), 453.0624490 ([M + Na]^+^).

*1-(4,5-Dihydro-1H-thieno[2′,3′:2,3]thiepino[4,5-c]pyrazole-3-carbonyl)-4-(2-methylphenyl)piperazine* (**6f**): White powder; yield: 38.0%; mp: 192–194 °C; IR (KBr, cm^−1^): υ 3442.6, 3264.4, 2919.3, 2851.2, 1601.8, 1492.0, 1474.7, 1442.8, 1383.7, 1223.1, 1152.0, 1026.2, 995.5, 861.7, 761.1, 691.0; ^1^H-NMR (600 MHz, CDCl_3_): δ 2.33 (s, 3H), 2.92–2.98 (m, 4H), 3.12 (t, 2H, *J* = 6.0 Hz), 3.48 (t, 2H, *J* = 6.0 Hz), 3.87–3.93 (m, 4H), 6.99–7.03 (m, 2H), 7.16–7.20 (m, 2H), 7.23 (d, 1H, *J* = 5.5 Hz), 7.27 (d, 1H, *J* = 5.5 Hz); ^13^C NMR (101 MHz, DMSO-*d*_6_): δ 163.16, 150.94, 145.69, 135.12, 132.45, 132.02, 130.92, 126.92, 126.65, 126.27, 123.29, 119.15, 116.70, 52.20, 51.56, 47.33, 42.02, 32.26, 29.47, 17.65; ESI-MS: m/z 409.0 ([M − H]^−^); HRMS (ESI): 411.130670 ([M + H]^+^), 433.112870 ([M + Na]^+^).

*1-(4,5-Dihydro-1H-thieno[2′,3′:2,3]thiepin[4,5-c]pyrazole-3-carbonyl)-4-(2-fluorophenyl)piperazine* (**6g**): White powder; yield: 28.0%; mp: 179–181 °C; IR(KBr, cm^−1^): υ 3439.4, 2918.7, 1611.8, 1500.0, 1440.3, 1384.7, 1342.1, 1286.6, 1236.6, 1201.2, 1147.9, 998.6, 884.5, 860.5, 747.8; ^1^H-NMR (600 MHz, CDCl_3_): δ 3.11 (t, 2H, *J* = 6.0 Hz), 3.10–3.12 (m, 4H), 3.48 (t, 2H, *J* = 6.0 Hz), 3.94–3.96 (m, 4H), 6.95–6.98 (m, 2H), 7.05–7.08 (m, 2H), 7.23 (d, 1H, *J* = 5.4 Hz), 7.27 (d, 1H, *J* = 5.4 Hz); ^13^C NMR (101 MHz, DMSO-*d*_6_): δ 163.04, 156.30, 153.88, 139.65, 139.57, 126.99, 126.32, 124.95, 122.97, 122.89, 119.75, 116.80, 116.18, 115.97, 50.98, 50.37, 46.81, 41.58, 32.23, 29.44; ESI-MS: *m*/*z* 415.3 ([M + H]^+^), 437.1 ([M + Na]^+^); HRMS (ESI): 415.106040 ([M + H]^+^), 437.087728 ([M + Na]^+^).

*1-(4,5-Dihydro-1H-thieno[2′,3′:2,3]thiepin[4,5-c]pyrazole-3-carbonyl)-4-(2-chlorophenyl)piperazine* (**6h**): White powder; yield: 38.0%; mp: 203–205 °C; IR(KBr, cm^−1^): υ 3441.5, 3214.1, 2920.0, 2851.1, 1601.9, 1500.3, 1477.4, 1443.6, 1384.1, 1336.5, 1286.9, 1237.1, 1202.8, 1146.5, 1025.4, 997.1, 861.8, 759.2, 689.2; ^1^H-NMR (600 MHz, CDCl_3_): δ 3.10–3.12 (br, 6H), 3.48 (t, 2H, *J* = 6.0 Hz), 3.95 (m, 4H), 7.02–7.04 (m, 3H), 7.22 (d, 1H, *J* = 5.4 Hz), 7.25 (d, 1H, *J* = 5.4 Hz), 7.37–7.39 (m, 1H); ESI-MS: *m*/*z* 431.0, 432.9 ([M + H]^+^); HRMS (ESI): 429.062045 ([M − H]^−^).

*1-(4,5-Dihydro-1H-thieno[2′,3′:2,3]thiepino[4,5-c]pyrazole-3-carbonyl)-4-(diphenylmethyl)piperazine* (**6i**): White powder; yield: 40.0%; m.p.: 138–139 °C; IR(KBr, cm^−1^): υ 3422.1, 3207.1, 2919.3, 2851.1, 1617.2, 1491.3, 1448.8, 1384.3, 1285.9, 1242.7, 1142.9, 995.3, 859.8, 747.1, 706.2; ^1^H-NMR (600 MHz, CDCl_3_): δ 2.41–2.46 (m, 4H), 3.08 (t, 2H, *J* = 6.0 Hz), 3.47 (t, 2H, *J* = 6.0 Hz), 3.73–3.78 (m, 4H), 5.25 (s, 1H), 7.19 (d, 2H, *J* = 7.2 Hz), 7.22 (d, 1H, *J* = 5.4 Hz), 7.26 (d, 1H, *J* = 5.4 Hz), 7.28 (t, 4H, *J* = 7.2 Hz), 7.41 (d, 4H, *J* = 7.2 Hz); ^13^C NMR (101 MHz, DMSO-*d*_6_): δ 162.95, 145.62, 142.45, 134.98, 132.43, 130.99, 128.62, 127.71, 127.01, 126.79, 126.33, 116.64, 74.74, 52.20, 51.46, 46.76, 41.61, 32.17, 29.67, 29.40; ESI-MS: *m*/*z* 487.2 ([M + H]^+^); HRMS (ESI): 487.162406([M + H]^+^), 509.144410 ([M + Na]^+^).

*(4,5-Dihydro-1H-thieno[2′,3′:2,3]thiepin[4,5-c]pyrazol-3-yl)(pyrrolidin-1-yl)methanone* (**6j**): Yellowish powder; yield: 49.0%; mp: 168–170 °C; IR (KBr, cm^−1^): υ 3263.2, 2920.4, 2875.7, 1595.8, 1526.9, 1492.2, 1470.4, 1432.4, 1382.8, 1339.4, 1224.1, 1007.6, 898.7, 861.2, 760.5, 688.5; ^1^H-NMR (600 MHz, CDCl_3_): δ 1.82 (t, 2H, *J* = 3.0 Hz), 1.93 (t, 2H, *J* = 3.0 Hz), 3.11 (t, 2H, *J* = 6.0 Hz), 3.33–3.37 (m, 4H), 3.59 (t, 2H, *J* = 6.0 Hz), 7.23 (d, 1H, *J* = 5.5 Hz), 7.25 (d, 1H, *J* = 5.5 Hz); ESI-MS: *m*/*z* 306.2 ([M + H]^+^), 328.1 ([M + Na]^+^); HRMS (ESI): 304.058839 ([M − H]^−^).

*(4,5-Dihydro-1H-thieno[2′,3′:2,3]thiepin[4,5-c]pyrazol-3-yl)(piperidin-1-yl)methanone* (**6k**): White powder; yield: 30.0%; mp: 190–191 °C; IR(KBr, cm^−1^): υ 3435.9, 3229.1, 2920.1, 2851.2, 1607.8, 1514.6, 1445.3, 1384.1, 1254.9, 1138.8, 1027.8, 989.4, 859.0, 774.5, 617.9; ^1^H-NMR (600 MHz, CDCl_3_): δ 1.25–1.27 (m, 2H), 1.56–1.69 (m, 4H), 3.11 (t, 2H, *J* = 6.0 Hz), 3.40–3.42 (m, 2H), 3.64–3.68 (m, 4H), 7.23 (1H, d, *J* = 5.4Hz), 7.24 (1H, d, *J* = 5.4 Hz); ESI-MS: *m*/*z* 320.1 ([M + H]^+^); HRMS (ESI): 318.074426 ([M − H]^−^).

*(4,5-Dihydro-1H-thieno[2′,3′:2,3]thiepin[4,5-c]pyrazol-3-yl)(morpholin-4-yl)methanone* (**6l**): White powder; yield: 52.0%; mp: 176–178 °C; IR(KBr, cm^−1^): υ 3439.5, 2920.5, 2851.6, 1611.7, 1434.2, 1271.5, 1242.1, 1194.6, 1115.5, 1024.6, 995.5, 861.4, 619.7; ^1^H-NMR (600 MHz, CDCl_3_): δ 3.11 (t, 2H, *J* = 6.0 Hz), 3.47 (t, 2H, *J* = 6.0 Hz), 3.74–3.75 (m, 4H), 3.81–3.82 (m, 4H), 7.22 (1H, d, *J* = 5.4 Hz), 7.25 (1H, d, *J* = 5.4 Hz); ESI-MS: *m*/*z* 322.0 ([M + H]^+^); HRMS (ESI): 320.053538 ([M − H]^−^).

### 3.2. Cell Proliferation Assay

The antiproliferative activities of target compounds were determined using a standard (MTT)-based colorimetric assay with gifitinib as the positive control.

A549 cells were cultured in its specific medium to the log phase before the experiment. Briefly, cell lines were seeded into 96-well microtiter plates (three wells per sample) at a density of 3 × 10^4^ cells/mL with 200 μL per well. After 24 h, exponentially growing cells were exposed to the indicated compounds at final concentrations (80 μM, 24 μM, 8 μM, 2.4 μM, 0.8 μM, and 0.24 μM). Gifitinib were used as the positive controls. After 24 h, cell survival was determined by the addition of an MTT solution (20 μL of 5 mg/mL MTT in PBS) to each well and the plates were incubated at 37 °C for a further 4 h. After 4 h, the medium was completely removed and 50 μL DMSO was added to each well. Optical absorbance was measured at 562 nm using an ELISA Reader. Survival ratios are expressed in percentages with respect to untreated cells. IC_50_ values were determined in least three independent experiments.

### 3.3. Molecular Docking

Molecular docking of compound **6g** and the positive control gefitinib into the three-dimensional and two-dimensional EGFR structure (4HJO.pdb, downloaded from the PDB) was carried out using the Molegro Virtual Docker 2008 (version 3.0) as implemented through the DS Viewer Pro (copyright©2002 Accelrys Inc., San Diego, CA, USA).

## 4. Conclusions

In the present work, a novel series of 4,5-dihydro-1*H*-thieno [2′,3′:2,3]thiepino[4,5-c]pyrazole-3-carboxamide derivatives have been designed and synthesized, and their biological activities were also evaluated as potent EGFR inhibitory. Biological activity evaluation showed that the compound **6g** demonstrated the most potent inhibitory activity (IC_50_ = 9.68 ± 1.95 μmol·L^−1^ for A549), which could have the potential to be developed for antiproliferative agents against cancer cells. A structure-activity relationship was clearly discernible. Docking simulation was performed to position compound **6g** into the EGFR to determine the probable binding model. The analysis of the binding conformation of **6g** demonstrated that the compound **6g** was stabilized by hydrogen bonds between the nitrogen atom of pyrazole and MET769 respectively. In summary, it is hoped that these derivatives could be used as potential lead compounds for further research.

## Figures and Tables

**Table 1:** IC_50_ values of the target compounds against A549 and HepG2.

No.	Substituents	IC_50_ (μM)	
		A549	HepG2
**6a**	4-methylpiperazin-1-yl	23.44 ± 3.32	>200
**6b**	4-phenylpiperazin-1-yl	21.38 ± 14.39	>200
**6c**	4-(4-methylphenyl)piperazin-1-yl	11.03 ± 1.96	>200
**6d**	4-(4-methoxyphenyl)piperazin-1-yl	13.13 ± 1.21	>200
**6e**	4-(4-fluorophenyl)piperazin-1-yl	92.71 ± 23.90	>200
**6f**	4-(2-methylphenyl)piperazin-1-yl	3.79 ± 13.39	>200
**6g**	4-(2-fluorophenyl)piperazin-1-yl	9.68 ± 1.95	>200
**6h**	4-(2-chlorophenyl)piperazin-1-yl	28.68 ± 11.71	>200
**6i**	4-(diphenylmethyl)piperazin-1-yl	104.98 ± 44.66	>200
**6j**	pyrrolidin-1-yl	25.98 ± 8.00	>200
**6k**	piperidin-1-yl	27.94 ± 13.79	>200
**6l**	morpholin-4-yl	12.62 ± 14.76	>200
Gifitinib		8.58 ± 1.65	>200
